# Emerging Roles of Post-Translational Modifications in Metabolic Homeostasis and Type 2 Diabetes

**DOI:** 10.3390/ijms262311552

**Published:** 2025-11-28

**Authors:** Yong Kyung Kim, Hyeongseok Kim

**Affiliations:** 1 Institute of Medical Science, College of Medicine, Yeungnam University, Daegu 42415, Republic of Korea; yongkyung.kim@yu.ac.kr; 2 Department of Biochemistry, College of Medicine, Chungnam National University, Daejeon 35015, Republic of Korea; 3 Department of Medical Science, College of Medicine, Chungnam National University, Daejeon 35015, Republic of Korea; 4 Biomedical Research Institute, Chungnam National University Hospital, Daejeon 35015, Republic of Korea

**Keywords:** post-translational modification, insulin sensitivity, metabolic disorder, diabetes mellitus

## Abstract

Post-translational modifications (PTMs) provide an integrated regulatory layer that couples nutrient and hormonal signals to whole-body energy homeostasis across metabolic organs. PTMs modulate protein activity, localization, stability, and metabolic networks in a tissue- and state-specific manner. Through network remodeling, PTMs integrate receptor signaling with chromatin and organelle function and align transcriptional control with mitochondrial function, proteostasis, and membrane trafficking. PTM crosstalk connects kinase cascades, nutrient-sensing pathways, and ubiquitin-family modifiers to orchestrate gluconeogenesis, lipolysis, glucose uptake, thermogenesis, and insulin secretion in response to nutrient cues. The metabolic state regulates PTM enzymes through changes in cofactors, redox tone, and compartmentalization, and PTM-dependent changes in transcription and signaling feedback to metabolic tone. In obesity and diabetes, dysregulated post translational modification networks disrupt insulin receptor signaling, disturb organelle quality control, and impair beta cell function, which promotes insulin resistance and beta cell failure. Consequently, PTMs organize metabolic information flow and modulate tissue responses to overnutrition and metabolic stress. A systems-level understanding of PTMs clarifies mechanisms of whole-body energy homeostasis and supports the discovery of new therapeutic targets in metabolic disease.

## 1. Introduction

Diabetes develops when the body cannot effectively control blood glucose levels, due to issues with insulin production in the beta cells or inadequate responses to insulin in target tissues. Both genetic predisposition and environmental factors contribute to its onset [1,2]. Pancreatic beta cells are central to glycemic control, and their loss or dysfunction leads to diabetes [3]. Type 1 diabetes is a disease caused by immune-mediated loss of insulin-producing beta cells, resulting in a lifelong intrinsic inability to maintain glucose homeostasis [2,4]. Type 2 diabetes is caused by a complex combination of genetic and environmental factors and is associated with obesity, insulin resistance, and islet dysfunction, including impaired glucose-stimulated insulin secretion (GSIS) [1,2].

Post-translational modifications (PTMs) are chemical or enzymatic changes that occur in proteins after translation, allowing them to be covalently altered either reversibly or irreversibly [5,6]. Modifications such as phosphorylation, methylation, ubiquitination, glycosylation, acylation, nitrosylation, and SUMOylation can alter protein stability, localization, and activity. These modifications affect various cellular processes and may disrupt normal cell function [7,8,9]. Traditional approaches to studying PTMs involve mass spectrometry (MS)-based proteomics and the use of antibodies that specifically recognize individual PTMs [10,11]. Growing evidence suggests that PTMs enhance protein diversity by modifying their structure, localization, and function. These modifications are crucial in numerous physiological and pathological processes, including cell division, apoptosis, regulation of transcription and translation, signal transduction, and immune responses [12,13,14].

In recent years, PTMs have emerged as central regulators of metabolic physiology. PTM networks regulate metabolic signaling and align gene expression with the metabolic state. Advances in multi-omics and quantitative proteomics continue to reveal extensive PTM crosstalk and context-dependent remodeling that track with metabolic state and disease risk. This review provides an overview of the major types of PTMs and their functional significance in metabolic regulation. We emphasize the roles and mechanisms of PTMs in disorders of glucose metabolism. This review aims to offer valuable insights into potential therapeutic approaches for managing these metabolic disorders.

## 2. Pathophysiology of Type 2 Diabetes

### 2.1. Type 2 Diabetes

Type 2 diabetes is one of the most prevalent chronic diseases worldwide and represents a major cause of morbidity, mortality, and health care expenditure [15]. It is a heterogeneous metabolic disease characterized by chronic hyperglycemia due to a combination of insulin resistance and inadequate insulin secretion. Type 2 diabetes is strongly associated with obesity, sedentary lifestyle, and aging, and its prevalence continues to rise in both developed and developing countries. The major drivers are insulin resistance in peripheral tissues such as the liver, adipose tissue, and skeletal muscle, and progressive beta cell dysfunction. Diverse factors, including low-grade inflammation in metabolic organs, oxidative and ER stress, and mitochondrial defects, amplify these drivers [16,17,18].

Persistent hyperglycemia and associated metabolic abnormalities lead to a broad spectrum of complications. Microvascular injury underlies diabetic retinopathy with visual loss, nephropathy with progression to chronic kidney disease and end-stage renal disease, and peripheral and autonomic neuropathy with pain, sensory loss, and impaired autonomic function. Macrovascular complications include coronary artery disease, stroke, and peripheral arterial disease, which increase the risk of myocardial infarction, heart failure, limb ischemia, and amputations. These complications impose a substantial burden on quality of life, functional capacity, and survival. Beyond these vascular complications, non-vascular complications such as fatty liver disease, cognitive impairment, bone fragility, and increased cancer risk are also gaining attention as important contributors to the overall burden of diabetes [19,20,21].

Type 2 diabetes is clinically heterogeneous, with variation in the relative contributions of insulin resistance and beta cell failure, patterns of obesity, and rates and profiles of complication development. Current therapies rarely achieve durable remission, and most individuals require lifelong management to limit disease progression and its complications. A deeper understanding of the mechanisms that link insulin resistance, beta cell failure, and end-organ injury and the identification of new therapeutic targets are essential for the development of more effective and disease-modifying therapies.

### 2.2. Insulin Resistance and Beta Cell Compensation

**Figure 1:**
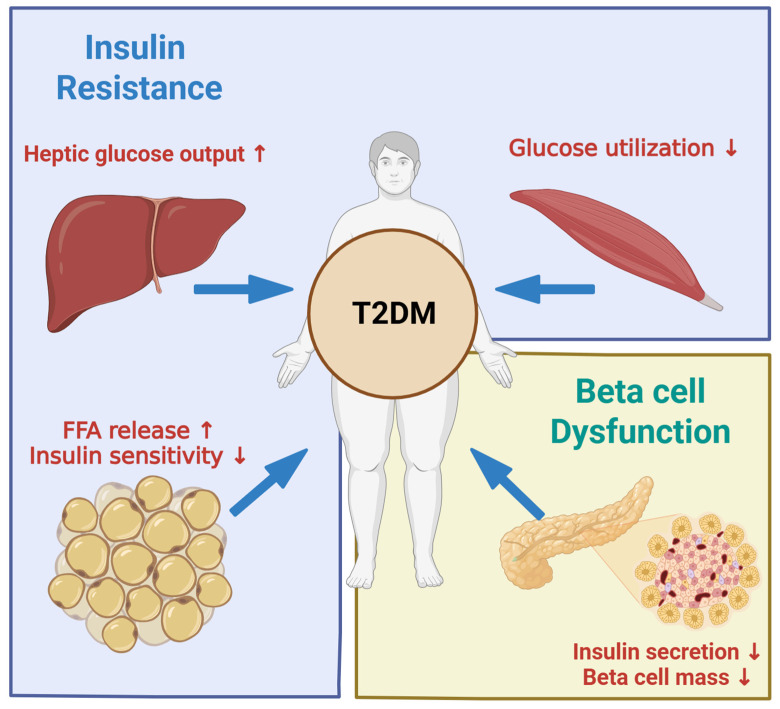
Pathophysiology of type 2 diabetes. Created in BioRender. Kim, Y. (2025) BioRender.com/i32lzrh.

Insulin resistance is a central feature in the pathogenesis of type 2 diabetes and refers to an impaired response to insulin in target tissues such as the liver, skeletal muscle, and adipose tissue (Figure 1). In the liver, impaired insulin action fails to suppress gluconeogenesis and glycogenolysis, which elevates hepatic glucose output, especially in the fasting state. In skeletal muscle, this defect lowers GLUT4 translocation and glucose uptake. In adipose tissue, reduced insulin sensitivity enhances lipolysis and increases the release of free fatty acids into the circulation. These fatty acids accumulate in the liver and skeletal muscle as toxic lipid intermediates and activate stress kinases, which further disrupt insulin receptor signaling [22,23]. The development of insulin resistance reflects a complex interaction among nutrient excess, ectopic lipid deposition, low-grade inflammation, mitochondrial dysfunction, and genetic susceptibility. Obesity and chronic overnutrition promote adipose tissue expansion and increase the secretion of proinflammatory adipokines, which in turn recruit immune cells that produce cytokines such as TNF-α and IL-6. These mediators, together with lipotoxic molecules, activate stress pathways and further impair insulin receptor signaling. Mitochondrial defects reduce oxidative capacity and favor accumulation of lipid metabolites. Hormonal factors such as altered adipokine and hepatokine profiles also modulate systemic insulin sensitivity [24,25,26].

In the early stages of insulin resistance, pancreatic beta cells initiate a compensatory response that maintains near-normal glucose levels. This process, often termed beta cell compensation, involves both quantitative and qualitative adaptations. Quantitative changes include an increase in beta cell mass through enhanced proliferation. Qualitative adaptations involve augmented insulin biosynthesis and strengthen GSIS. Experimental and clinical studies indicate that insulin resistance from obesity, pregnancy, or high-fat feeding is usually accompanied by expanded beta cell mass and hyperinsulinemia [27,28]. In rodent models, beta cell area can increase several-fold under sustained metabolic load [29]. These changes reflect activation of cell cycle regulators and suppression of proapoptotic signals. Growth factor and hormone pathways that signal through AKT, JAK-STAT, MAPKs, and mTOR support beta cell survival and proliferation in this context [30]. In addition, transcription factors including PDX1 and FOXM1 maintain the proliferative and secretory phenotype required for compensation [31,32]. However, beta cell compensation has finite limits. When insulin resistance is severe, prolonged, or combined with genetic susceptibility, compensatory mechanisms fail. The capacity for beta cell compensation explains why many individuals with obesity or insulin resistance maintain normal glucose tolerance for years, while others progress rapidly to type 2 diabetes. Differences in beta cell mass expansion, survival signaling, and stress resilience contribute to this heterogeneity.

### 2.3. Beta Cell Failure

Beta cell failure is a key process in the natural history of type 2 diabetes and develops when beta cells can no longer adequately compensate for insulin resistance and glycemic control begins to fail. When metabolic stress exceeds adaptive capacity, beta cells lose the ability to secrete sufficient insulin for the level of insulin resistance. This failure reflects a combination of reduced beta cell mass, impaired stimulus–secretion coupling, loss of mature identity, and increased vulnerability to stress [33,34].

ER stress has a central position in beta cell failure. Beta cells synthesize and fold large amounts of proinsulin under normal conditions. Increased secretory demand induces further expansion of the endoplasmic reticulum workload. Misfolded proteins accumulate and activate the unfolded protein response. Transient activation can restore proteostasis. However, chronic activation of PERK, IRE1, and ATF6 pathways suppresses global translation and favors proapoptotic transcription programs. CHOP and related factors promote mitochondrial outer membrane permeabilization and caspase activation. As these pathways progress, beta cell survival falls and islet architecture deteriorates [35,36].

Mitochondrial dysfunction and oxidative stress are tightly linked components of beta cell failure. Dysfunctional mitochondria produce excessive reactive oxygen species. Beta cells have relatively low expression of antioxidant enzymes such as catalase and glutathione peroxidase, which makes them highly susceptible to oxidative damage. Reactive oxygen species modify proteins, lipids, and DNA, alter key signaling molecules, and amplify ER stress. Defective mitophagy allows damaged mitochondria to persist and maintain high oxidative stress, which accelerates apoptosis and loss of functional beta cell mass [37,38].

Loss of beta cell identity is another key feature of failure. Under sustained metabolic and inflammatory stress, expression of lineage-defining transcription factors such as PDX1, NKX6.1, and MAFA decreases. Genes that characterize mature beta cells show reduced transcription. In parallel, genes characteristic of progenitor cells or other islet cell types are aberrantly re-expressed. Some beta cells enter a dedifferentiated state with low insulin content and altered hormone expression. Although these cells are not dead, they do not contribute effectively to insulin secretion. This phenomenon reduces functional beta cell mass even when cell number has not yet fallen dramatically [39,40,41].

Collectively, these findings indicate that the pathogenesis of diabetes is influenced by highly integrated intracellular signaling networks and diverse molecular alterations. Signaling pathways that regulate cell cycle progression, cell survival and apoptosis, the unfolded protein response, mitochondrial quality control, redox balance, and lineage-determining transcription factors all contribute to the balance between adaptive beta cell expansion and functional decline, and to the degree of insulin resistance in the liver, skeletal muscle, and adipose tissue. The activity and crosstalk of these pathways are tightly controlled by PTMs, which rapidly tune protein stability, localization, and interaction with partner molecules. Dynamic changes in PTM patterns are therefore likely to be critical molecular switches that determine whether insulin resistant tissues and pancreatic beta cells remain in a compensated state or progress toward overt beta cell failure and type 2 diabetes. Current evidence supports a significant role for PTMs as key modulators in the regulation of metabolic homeostasis and in the pathogenesis of diabetes. In this review, we discuss how specific PTMs in insulin target tissues and pancreatic beta cells contribute to the development and progression of diabetes and consider their potential as therapeutic targets.

## 3. Post-Translational Modifications

Proteins rarely remain in their nascent form after translation. Instead, they undergo a wide spectrum of post-translational modifications that remodel their function, stability, and cellular localization. Post translational modifications are covalent chemical changes that occur on proteins after translation and expand the functional diversity of the proteome beyond what is encoded by the genome. Since the recognition of phosphorylation as a signaling switch and the proposal of the histone code, post-translational modifications have been regarded as a dynamic interface between metabolism, signaling, and gene regulation [42,43].

These modifications include phosphorylation, ubiquitination, acetylation, methylation, SUMOylation, glycosylation, lipidation, redox-based modifications, and many others. Specific amino acid residues such as serine, threonine, tyrosine, lysine, arginine, cysteine, and asparagine serve as major PTM sites. Addition or removal of these chemical groups can alter protein conformation, catalytic activity, subcellular localization, stability, and affinity. In this way, PTMs integrate extracellular and intracellular signaling networks into rapid and spatially restricted changes in protein function without the need for new protein synthesis. PTMs are written, erased, and interpreted by specific enzymes and binding domains that act as dynamic regulatory modules. Kinases, acetyltransferases, ubiquitin ligases, and related enzymes add modifications, whereas phosphatases, deacetylases, deubiquitinases, and desumoylating proteases remove them. Reader proteins that contain domains such as SH2, 14-3-3, bromodomains, or ubiquitin binding motifs recognize specific PTMs and assemble downstream signaling complexes. Individual proteins often carry multiple PTMs at distinct sites, and these modifications can cooperate or compete with one another, which creates PTM patterns that resemble a regulatory code [44,45,46].

This layered control is particularly important in metabolic pathways and stress responses, where PTMs fine-tune insulin signaling, organelle quality control, and cell fate decisions that influence the development and progression of insulin resistance and diabetes. Within metabolic organs, PTMs serve as sensors of nutrient flux and energy. Phosphorylation rapidly relays insulin and AMPK signaling, adjusting glucose uptake, glycogen synthesis, and lipid oxidation [47]. Acetylation, governed by lysine acetyltransferases and deacetylases such as Sirtuins, reflects acetyl-CoA abundance and links nutrient state to mitochondrial efficiency and transcriptional control [48]. Ubiquitination and related modifiers regulate enzyme turnover and insulin receptor stability, ensuring proper termination of signaling cascades [49]. Meanwhile, O-GlcNAcylation acts as a glucose-responsive modification that modulates both cytosolic enzymes and transcription factors, frequently antagonizing phosphorylation to fine-tune insulin sensitivity [50].

The interplay among these modifications establishes a multilayered regulatory system. Crosstalk between phosphorylation, acetylation, and ubiquitination determines whether energy is stored or expended, while histone acylation integrates intermediary metabolites into epigenomic regulation of metabolic genes [51]. Under physiological conditions, this network preserves energy homeostasis across the liver, skeletal muscle, and adipose tissue. However, chronic nutrient excess—characteristic of obesity—distorts PTM dynamics. Elevated cytosolic acetyl-CoA enhances global protein acetylation, suppressing fatty-acid oxidation; persistent hyperglycemia increases O-GlcNAcylation of key signaling proteins, desensitizing insulin pathways; and aberrant phosphorylation of IRS and AKT further amplifies insulin resistance [52,53].

These molecular alterations converge in type 2 diabetes, where PTM imbalance underlies impaired insulin signaling and metabolic inflexibility. In the liver, hyperacetylation of gluconeogenic enzymes sustains hepatic glucose output despite insulin presence. In skeletal muscle, reduced AMPK-dependent phosphorylation weakens glucose uptake and mitochondrial biogenesis. Adipose tissue displays excessive ubiquitin-mediated degradation of insulin-sensitive receptors, promoting systemic resistance to insulin action. Oxidative and inflammatory stress introduce further layers—nitrosylation, carbonylation, and SUMOylation—that aggravate mitochondrial dysfunction and lipid peroxidation [54].

Emerging proteomic and metabolomic studies reveal that type 2 diabetes involves not a single defective pathway but a global reorganization of the PTM landscape. Understanding how nutrient-responsive modifications shape metabolic enzyme activity, chromatin architecture, and hormonal signaling opens new therapeutic directions. Pharmacological modulation of PTM enzymes—such as Sirtuin activators, HDAC inhibitors, and O-GlcNAc cycling regulators—represents a promising strategy to restore metabolic flexibility and counteract insulin resistance.

## 4. Post-Translational Modifications in Type 2 Diabetes

**Figure 2:**
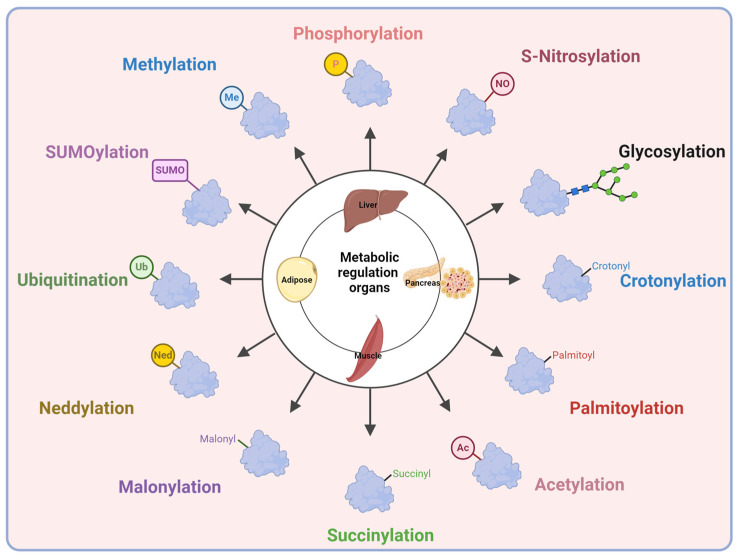
Post-translational modification in metabolic organs. Created in BioRender. Kim, Y. (2025) BioRender.com/iizhezf.

**Figure 3:**
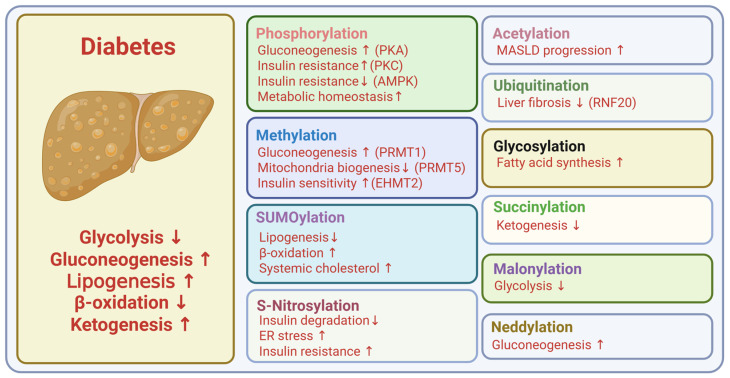
Metabolic roles of PTMs in liver. Created in BioRender. Kim, H. (2025) BioRender.com/bxe5ipm.

**Figure 4:**
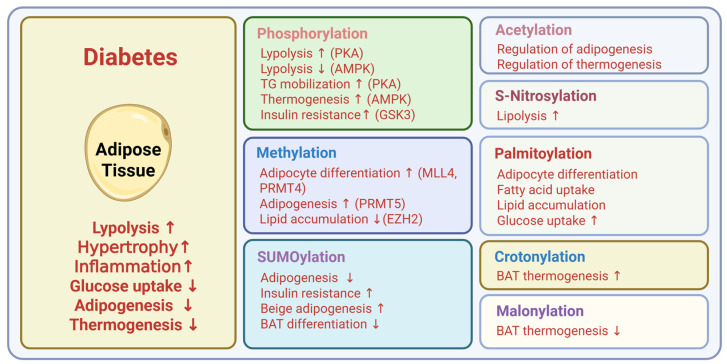
Metabolic roles of PTMs in adipose tissue. Created in BioRender. Kim, H. (2025) BioRender.com/iaawf9v.

**Figure 5:**
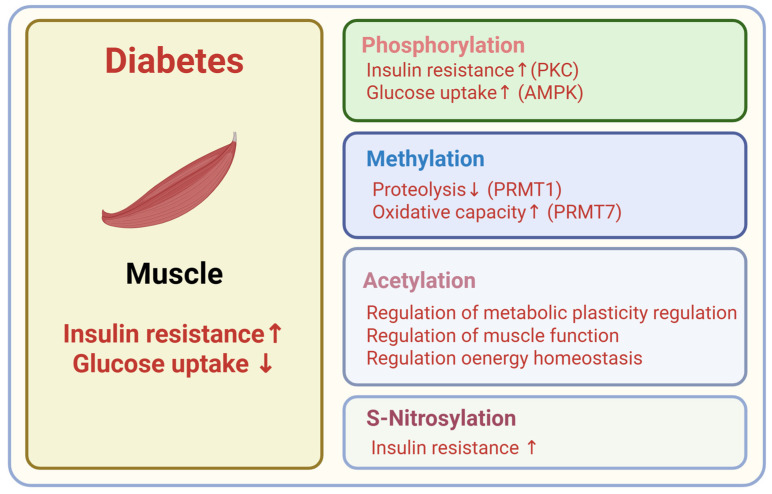
Metabolic roles of PTMs in skeletal muscle. Created in BioRender. Kim, H. (2025) BioRender.com/rkh9dss.

**Figure 6:**
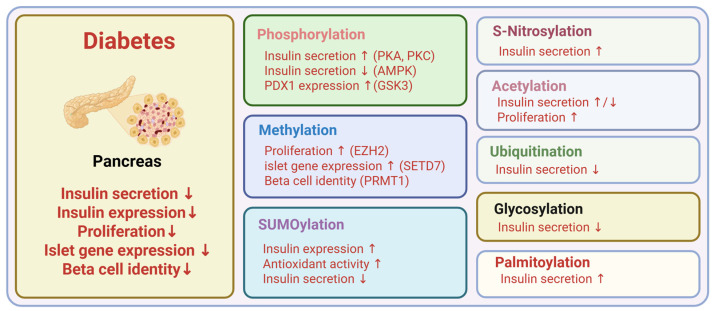
Metabolic roles of PTMs in pancreatic beta cells. Created in BioRender. Kim, Y. (2025) BioRender.com/i32lzrh.

The liver, adipose tissue, skeletal muscle, and pancreatic beta cells are central regulators of whole-body energy metabolism and represent the major sites of insulin action and secretion (Figure 2). Hepatic PTMs modulate insulin signaling pathways, gluconeogenic enzymes, and lipid metabolism, which directly influence hepatic glucose production (Figure 3). PTMs in adipose tissue regulate adipokine secretion, lipolysis, and inflammatory signaling that feed back on systemic insulin sensitivity (Figure 4). Within skeletal muscle, PTMs affect insulin-stimulated glucose transport, cytoskeletal organization, and mitochondrial function, which are key determinants of peripheral glucose disposal (Figure 5). In pancreatic beta cells, PTMs fine-tune GSIS, ER and mitochondrial stress responses, and the maintenance of beta cell identity and survival (Figure 6). Focusing on PTMs in these four tissues is therefore essential to understand how nutrient and hormonal cues are converted into molecular signals that support metabolic homeostasis or promote progression to diabetes.

### 4.1. Phosphorylation

**Figure 7:**
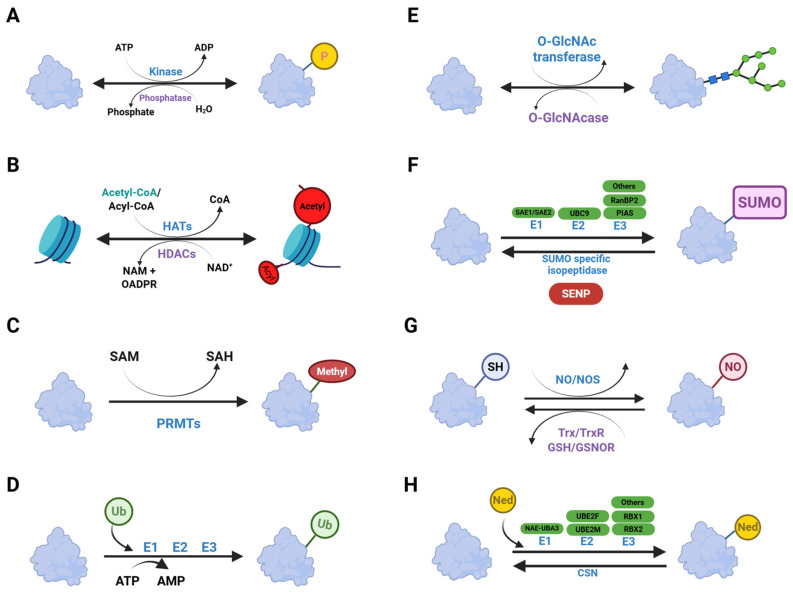
Mechanism of post-translational modification. (**A**) Phosphorylation. (**B**) Acetylation. (**C**) Methylation. (**D**) Ubiquitination. (**E**) Glycosylation. (**F**) SUMOylation. (**G**) S-Nitrosylation. (**H**) Neddylation. Created in BioRender. Kim, Y. (2025) BioRender.com/19pdp1n.

Phosphorylation is the covalent attachment of a phosphate group to specific amino acid residues, catalyzed by protein kinases. This modification regulates protein activity and interactions [55]. Phosphorylation is broadly involved in various regulatory processes, such as membrane transport, protein degradation, modulation of enzyme activity, and protein-protein interactions [55,56]. Protein phosphorylation is among the most prevalent and functionally significant PTMs [56,57]. This modification is reversible and tightly regulated by the coordinated actions of protein kinases and phosphatases (Figure 7A).

Protein kinase A (PKA) and protein kinase C (PKC) couple nutrient and hormonal cues to metabolic programs across the liver, adipose tissue, skeletal muscle, and pancreatic beta cells. PKA is the principal effector of cAMP downstream of glucagon and catecholamine signaling and promotes hepatic gluconeogenesis [58,59,60]. In adipose tissue, PKA phosphorylates perilipin and hormone-sensitive lipase to stimulate lipolysis and mobilize stored triacylglycerol [61]. PKA signaling also supports thermogenic programming in brown and beige fat in part through pathways that induce PGC-1α and UCP1 [62,63]. In pancreatic beta cells, PKA enhances Ca^2+^ entry, vesicle priming, and exocytotic competence to potentiate GSIS [64]. PKC isoforms are organized into conventional, novel, and atypical classes with distinct lipid and Ca^2+^ sensitivities and they integrate diacylglycerol and lipid-derived signals with metabolic control [65]. Lipid-activated PKCθ in skeletal muscle and PKCε in liver impair insulin receptor signaling and contribute to insulin resistance in nutrient excess [66,67]. Hepatic PKCδ and PKCβ promote steatotic and lipogenic programs and are linked to dysregulated lipid handling and VLDL-related adaptations [68,69,70]. In adipose tissue, PKC signaling modulates lipolysis, adipogenesis, and inflammatory tone, and in beta cells PKC activity regulates the kinetics of biphasic insulin secretion [65,71].

AMP-activated protein kinase (AMPK) is a central energy sensor that restores metabolic homeostasis by promoting catabolic pathways and restraining anabolic programs across liver, adipose tissue, skeletal muscle, and pancreatic beta cells [72]. AMPK suppresses mTORC1 through phosphorylation of TSC2 and Raptor and it promotes autophagy via direct phosphorylation of ULK1 [73,74]. In the liver, AMPK coordinates lipid and glucose metabolism to support insulin sensitivity and limit steatosis. Pharmacologic or genetic activation of hepatic AMPK improves metabolic homeostasis in preclinical models, whereas reduced AMPK activity under nutrient excess is associated with lipogenic drive and hepatocellular stress [75,76,77,78]. In skeletal muscle, AMPK activation increases glucose uptake through GLUT4 translocation during contraction or pharmacologic stimulation [79]. In adipose tissue, AMPK supports thermogenic programming and browning while maintaining mitochondrial homeostasis in brown and beige fat [80,81]. AMPK also restrains adipocyte lipolysis by phosphorylating hormone-sensitive lipase and counteracting PKA-dependent activation [82,83]. In pancreatic beta cells, glucose suppresses AMPK activity, and AMPK activation can restrain insulin secretion and affect beta cell proliferation and survival [84,85,86].

Recent advances in phosphoproteomics now enable system-wide mapping of insulin-responsive phosphorylation with tissue and disease resolution. In pancreatic islets, integrated proteomic and phosphoproteomic analyses have uncovered substantial remodeling of critical signaling pathways in islets affected by type 2 diabetes [87]. In adipose tissue, profiling across insulin-resistance models shows attenuation of canonical insulin-responsive sites. MARK2/3 and GSK3 dysregulation is a shared hallmark, and acute GSK3 inhibition partially restores insulin sensitivity [88]. In human skeletal muscle, personalized phosphoproteomics identifies the deubiquitinase MINDY1 as a modulator of insulin action that alters insulin-stimulated signaling and glucose uptake [89].

It has long been proposed that abnormal phosphorylation of islet proteins plays a key role in the onset and progression of type 2 diabetes. Glycogen synthase kinase 3 (GSK3) is a critical regulator of the beta cell transcription factor PDX1, which is essential for glucose sensing and insulin secretion [87,90]. Maintenance of ER homeostasis is vital for proper beta cell function, and its disruption induces ER stress and activates the unfolded protein response (UPR) [91]. Protein kinase R–like ER kinase (PERK), a key UPR component, senses ER stress and phosphorylates the α-subunit of eukaryotic translation initiation factor 2 (eIF2α), resulting in a reduction in global protein synthesis [91,92].

### 4.2. Acetylation

Histone acetylation is primarily regulated by the opposing actions of histone acetyltransferases (HATs) and histone deacetylases (HDACs). Three main families of HATs—GNAT, MYST, and p300/CBP—have been identified, all of which utilize acetyl-coenzyme A (Acetyl-CoA) as the donor of the acetyl group. Some of the studies have demonstrated that proper regulation of histone acetylation is crucial for the proliferation and functional maintenance of pancreatic beta cells [93]. HDACs are metalloenzymes categorized into three major classes, based on their sequence similarity to yeast deacetylase proteins [94,95,96,97,98,99]. Class I HDACs, which include HDAC1, HDAC2, HDAC3, and HDAC8, are homologous to the yeast transcriptional regulator Rpd3 (reduced potassium dependency 3). Class II HDACs are further subdivided into class IIa—comprising HDAC4, HDAC5, HDAC7, and HDAC9—and class IIb, which includes HDAC6 and HDAC10, both sharing structural similarities with yeast Hda1 (histone deacetylase I) (Figure 7B).

Skeletal muscle, the largest organ system in mammals and the primary site of glucose disposal and energy metabolism, plays a central role in maintaining systemic homeostasis [100]. Beyond its contractile functions, skeletal muscle acts as a dynamic metabolic hub that integrates nutrient sensing, energy expenditure, and inter-organ communication [101]. Epigenetic regulation, particularly histone acetylation, has emerged as a fundamental mechanism by which chromatin architecture and transcriptional networks are remodeled to support these diverse functions [102]. The balance between histone acetyltransferases and deacetylases dynamically modulates acetylation states, thereby shaping chromatin accessibility and enabling context-specific transcriptional programs that govern skeletal muscle metabolic plasticity [103]. Such regulation is critical during physiological adaptations, including exercise-induced remodeling, fasting–feeding cycles, and thermogenic responses, as well as in pathological contexts such as insulin resistance, sarcopenia, and type 2 diabetes. By linking environmental cues to gene expression, histone acetylation serves as a key epigenomic mechanism that integrates metabolic signals with skeletal muscle function and systemic energy homeostasis [104,105].

Over the past decade, the escalating prevalence of obesity and its related metabolic disorders has emerged as a critical global health concern. To address the heightened morbidity and mortality associated with the obesity epidemic, a wide range of therapeutic strategies have been explored. Recent advances in adipocyte biology have underscored the potential of thermogenic adipose tissue as a powerful modulator of systemic metabolism and a promising target for alleviating metabolic dysfunction. Concurrently, epigenetic research has revealed that histone acetylation plays a pivotal role in regulating adipogenesis and thermogenesis, thereby highlighting the essential functions of HATs and HDACs in controlling metabolism and maintaining systemic energy homeostasis [106,107].

In the liver, acetylation and deacetylation modifications at histone sites H3K9, H3K27, and H4K8 are primarily associated with the regulation of protein expression and silencing. Histone acetylation can promote the progression of Metabolic dysfunction-associated steatotic liver disease (MASLD) by enhancing ChREBP-mediated lipogenesis and facilitating fat accumulation through pathways involving NR4A1, LncRNA-NEAT1, and SCD [108,109,110]. Conversely, deacetylases such as Sirt1 and Sirt6 inhibit MASLD progression by suppressing PNPLA3-associated oxidative stress and mitigating fat accumulation linked to Smad3 signaling [111,112,113]. Although Sirt1 and Sirt6 were initially characterized as NAD-dependent histone deacetylases, accumulating evidence indicates that members of the Sirtuin family also deacetylate non-histone substrates and in this way modulate hepatic fatty acid oxidation, lipid synthesis, and triglyceride storage. More broadly, acetylation and deacetylation of non-histone proteins, including metabolic enzymes, transcription factors, and organelle-associated proteins, represent key mechanisms in MASLD progression and have attracted considerable attention as potential therapeutic targets [114].

Numerous studies have explored the function of HDACs in pancreatic beta cells, yielding mixed findings. However, inhibition of HDACs in cell lines and rodent islets has been shown to protect beta cells from cytokine-induced damage and promote their proliferation [115,116,117,118]. Moreover, mice with an inducible, beta cell-specific deletion of HDAC3 using the MIP-CreERT system exhibited improved glucose tolerance and enhanced insulin secretion [119]. In contrast, mice with a constitutive beta-cell-specific deletion of HDAC3 driven by the Rip-Cre system showed impaired beta-cell function [120]. The varying outcomes of these studies suggest that HDACs play a more intricate role than previously recognized. A dynamic equilibrium between HAT and HDAC activities may serve as a molecular rheostat, enabling cells to sense metabolic states and adjust their responses accordingly.

### 4.3. Methylation

Protein methylation is a widespread post-translational modification in which monomethyl, dimethyl, or trimethyl groups are added to lysine and arginine residues, modulating protein conformation, interaction surfaces, and chromatin binding. Lysine methylation is written by SET-domain lysine methyltransferases and erased by FAD-dependent KDM1A/B and Fe^2+^- and 2-oxoglutarate–dependent JmjC dioxygenases, with KDM1A/B acting on mono- and dimethylated lysines but not trimethylated sites. Arginine methylation is catalyzed by protein arginine methyltransferases (PRMTs) and is regulated by PRMT activity and substrate turnover (Figure 7C) [121,122,123]. In metabolic tissues, protein methylation regulates transcriptional and signaling pathways fundamental to glucose and lipid homeostasis, including PDX1-dependent islet programs, AKT and FOXO signaling, PPARγ-driven adipogenesis, and mitochondrial biogenesis. Nutrient and hormonal cues remodel methylation by altering enzyme abundance, cofactor availability, and subcellular localization, coupling cellular redox and metabolic status to gene regulation.

Protein methylation contributes to hepatic glucose and lipid metabolism by regulating key transcriptional and signaling pathways. PRMT1 promotes hepatic glucose production through FoxO1-dependent transcription. Arginine methylation of FOXO factors antagonizes AKT-mediated phosphorylation and sustains FOXO activity. This modification links methylation to hepatic control of gluconeogenesis [124,125]. PRMT5 inhibition increases mitochondrial biogenesis, elevates PPARα and PGC-1α, and reduces PI3K–AKT signaling [126]. Hepatic G9a/EHMT2, a histone H3K9 mono- and dimethyltransferase, supports insulin signaling and maintains HMGA1 and insulin receptor expression. Loss of G9a lowers INSR and reduces AKT and GSK3β phosphorylation, whereas hepatic G9a restoration in db/db mice raises HMGA1 and improves glycemic control [127].

In adipose tissue, protein methylation regulates adipocyte differentiation, lipid storage, and systemic metabolism. MLL4 (KMT2D), an H3K4 mono- and dimethyltransferase, is required for enhancer activation during cell differentiation. Loss of MLL4 reduces H3K4me1 and H3K27ac as enhancers and blunts induction of lineage-specific genes [128]. CARM1 (PRMT4) is an arginine methyltransferase that modifies histone H3 and functions as a transcriptional coactivator. CARM1 cooperates with PPARγ at metabolic promoters to promote adipocyte differentiation, and its deficiency lowers lipid-metabolism gene expression, reduces brown fat development, and impairs adipogenesis [129]. PRMT5 promotes adipogenesis by inducing PPARγ2 and its target genes and, in white adipose tissue, regulates fatty-acid metabolism and lipid-droplet biogenesis [130,131]. EZH2, the catalytic subunit of Polycomb repressive complex 2 (PRC2), mediates H3K27 trimethylation and acts as a transcriptional repressor. In adipose tissue, EZH2 regulates adipocyte lipid metabolism independently of adipogenic differentiation, and genetic or pharmacologic EZH2 inhibition increases ApoE and enhances lipoprotein-dependent lipid accumulation in adipocytes [132].

Methylation influences muscle homeostasis and energy metabolism. PRMT1 maintains skeletal muscle integrity by counteracting a PRMT6–FOXO3 axis that drives autophagy and proteolysis [133]. PRMT7 sustains oxidative metabolism through a p38MAPK–ATF2–PGC-1α signaling axis, and Prmt7 deficiency lowers oxidative capacity and predisposes to age-related obesity [134].

Protein methylation regulates beta cell proliferation, identity, and insulin secretion. EZH2, the H3K27 methyltransferase of Polycomb repressive complex 2, represses the Ink4a/Arf locus in pancreatic beta cells. Beta cell–specific Ezh2 loss derepresses p16Ink4a and p19Arf, reduces proliferative capacity, and impairs regenerative responses in diabetic settings [135]. SETD7 (also known as SET7/9), an H3K4 methyltransferase, maintains euchromatin and supports transcription at islet-enriched genes in beta cells. It methylates PDX1 and enhances its transcriptional activity at target loci, reinforcing islet gene expression programs [136,137]. PRMT1 preserves mature beta cell identity by maintaining H4R3me2a-dependent chromatin accessibility at CTCF and beta cell transcription factor sites. Deletion of Prmt1 in beta cells rapidly depletes H4R3me2a, downregulates mature beta cell genes, and induces diabetes, which worsens under high-fat diet stress [138].

### 4.4. Ubiquitination

Ubiquitination is a common post-translational modification in which ubiquitin is covalently attached to target proteins. This process is facilitated by a cascade of three enzymes: E1 (ubiquitin-activating), E2 (ubiquitin-conjugating), and E3 (ubiquitin ligases). Ubiquitin (Ub) is a highly conserved 76-amino-acid protein and contains seven lysine residues (K6, K11, K27, K29, K33, K48, and K63), each capable of assembling distinct polyubiquitin chains with specific functional consequences (Figure 7D) [139,140]. Extensive research has demonstrated that ubiquitination plays a pivotal role in a wide range of physiological and pathological processes, including transcriptional regulation, cell proliferation, apoptosis, DNA damage repair, and immune system modulation [141,142]. A dynamic balance between ubiquitination and deubiquitination is essential for protein homeostasis and normal cellular function. Disruptions in this balance within the ubiquitin system have been linked to the development of various diseases [143,144].

In the context of MASLD progression, research on histone ubiquitination remains relatively limited, despite its crucial role in modulating chromatin architecture, recruiting effector proteins, and activating downstream chromatin regulatory pathways. Notably, overexpression of RNF20 has been shown to suppress the expression of IL-6, TNFα, and vascular endothelial growth factor A (VEGFA) through monoubiquitination of histone H2B at lysine 120. This modification effectively counteracts TGF-β–induced activation of hepatic stellate cells (LX-2) and attenuates liver fibrosis [120,145].

In the context of beta cell dysfunction and type 2 diabetes progression, a critical subset of proteins involved in insulin secretion is subject to regulation through ubiquitination-mediated degradation. Specifically, the ubiquitination-induced degradation of glucokinase impairs insulin secretion by reducing the production of glucose-6-phosphate [146]. The E3 ubiquitin ligase Hrd-1 promotes the ubiquitination and subsequent degradation of MafA in pancreatic beta cells, leading to its cytoplasmic accumulation. This mislocalization diminishes MafA’s nuclear activity and consequently reduces insulin secretion [147]. Somatostatin receptor subtype 5 (SSTR5) suppresses PDX-1 expression by downregulating Pdx-1 transcription and promoting post-translational ubiquitination of PDX-1, thereby leading to reduced insulin secretion [148]. In pancreatic beta cells, phosphorylation of PDX-1 at Thr11 by macrophage-stimulating 1 (MST1) promotes its ubiquitination and subsequent degradation, thereby impairing insulin secretion [9,149].

### 4.5. Glycosylation

Glycosylation is considered one of the most diverse post-translational modifications. It can occur enzymatically or through non-enzymatic glycation, where glucose in its aldehyde form reacts with lysine and arginine residues in proteins. These reactions can progress to form advanced glycation end products (AGEs), which play significant roles in aging and are particularly relevant in the context of diseases such as diabetes [150]. Protein glycosylation is a complex, multi-step process involving approximately 200 glycosyltransferase enzymes. These enzymes regulate which proteins are glycosylated, determine the specific sites of glycan attachment, and orchestrate the assembly of distinct glycan structures [150,151]. Research on glycosylation enzyme deficiencies in both animal models and human diseases has significantly deepened our understanding of the biological roles of protein glycosylation. These studies have shown that most glycosyltransferases are crucial for maintaining normal physiological functions in mammals [152,153,154].

Earlier studies indicated that elevated levels of O-GlcNAcylation—arising from increased expression of O-GlcNAc transferase (OGT) or inhibition of O-GlcNAcase (OGA)—may contribute to insulin resistance via the hexosamine biosynthetic pathway (HBP) (Figure 7E) [155]. O-GlcNAcylation of insulin receptor substrate 1 (IRS-1) has been shown to impair AKT signaling, thereby contributing to the development of insulin resistance [156]. Studies have shown that O-GlcNAcylation of PDK1 and AKT impairs the insulin signaling pathway, further contributing to insulin resistance [157,158]. High intracellular glucose levels enhance flux through the hexosamine biosynthetic pathway (HBP), resulting in increased protein O-GlcNAcylation [159]. This elevated O-GlcNAcylation in peripheral tissues, including pancreatic islets, has been linked to the pathogenesis of diabetes.

MASLD is a metabolic liver disorder closely linked to hepatic nutrient metabolism. Conditions such as obesity and type 2 diabetes can promote hepatic triglyceride accumulation, which serves as a key driver of MASLD development [160,161]. Furthermore, Metabolic dysfunction-associated steatohepatitis represents the most severe form of MASLD and is recognized as a critical precursor to the onset of cirrhosis and hepatocellular carcinoma (HCC). As a key nutrient sensor, O-GlcNAcylation modulates hepatic triglyceride accumulation by regulating upstream glucose uptake, downstream fatty acid synthesis, and additional metabolic pathways. It has been reported that O-GlcNAc modification of fatty acid synthase (FAS) enhances its interaction with the deubiquitinase ubiquitin-specific protease 2a (USP2A), thereby reducing ubiquitin-mediated degradation and increasing FAS expression, which promotes fatty acid synthesis [162]. Additionally, carbohydrate-responsive element-binding protein (ChREBP) and sterol regulatory element-binding protein 1c (SREBP1c), key regulators of FAS expression, are influenced indirectly by O-GlcNAcylation. Through modification of liver X receptors (LXRs), O-GlcNAcylation enhances the transcription of ChREBP and SREBP1c, thereby upregulating FAS expression and promoting fatty acid synthesis [163,164,165].

O-GlcNAcylation plays a pivotal role in maintaining glucose homeostasis and insulin sensitivity in skeletal muscle, thereby conferring metabolic plasticity that allows adaptation to variations in nutrient availability and physiological cues. However, the global patterns of O-GlcNAcylation in skeletal muscle are highly complex and are influenced by factors such as muscle fiber type, inactivity, rest, and exercise modalities, including type and intensity [166]. O-GlcNAc transferase (OGT) is the sole enzyme in the human genome responsible for attaching a single O-GlcNAc moiety to serine and threonine residues of target proteins. Metabolic homeostasis is closely intertwined with O-GlcNAc cycling, with the hexosamine biosynthesis pathway (HBP) providing UDP-GlcNAc as a substrate for OGT [50,167]. Notably, adipocyte-specific OGT has been reported to reactivate lipid desaturation, resulting in increased accumulation of endocannabinoids within adipose tissue. Nevertheless, it remains unclear whether OGT in adipocytes affects hepatic stellate cell (HSC) differentiation, particularly under obesity-prone conditions [168,169].

Pancreatic beta cells exhibit high levels of O-GlcNAc and its modifying enzyme O-GlcNAc transferase (OGT), and O-GlcNAcylation has been shown to be essential for proper beta cell function [159,170]. Notably, in Goto-Kakizaki (GK) rats—a model of type 2 diabetes—pharmacological enhancement of O-GlcNAcylation in pancreatic islets leads to impaired GSIS, highlighting the critical role of O-GlcNAcylation in the regulation of insulin secretion [171]. Since O-GlcNAcylation targets serine and threonine residues, it can potentially compete with phosphorylation at the same sites, including those modified by PKA [172,173].

### 4.6. Acylation

**Figure 8:**
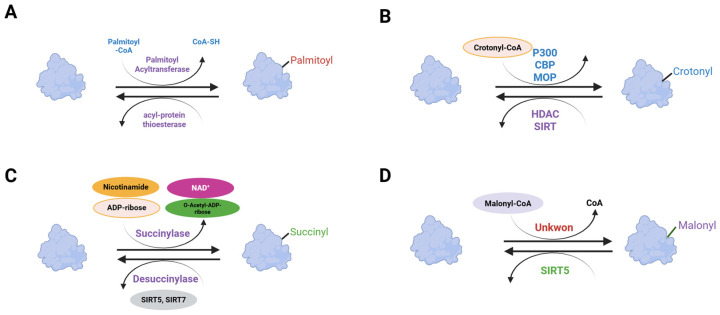
Mechanism of protein acylation. (**A**) Palmitoylation. (**B**) Crotonylation. (**C**) succinylation. (**D**) malonylation. Created in BioRender. Kim, Y. (2025) BioRender.com/4vnlcle.

Protein acylation is the covalent attachment of acyl groups that couples nutrient status to signaling, gene regulation, and membrane dynamics. S-acylation adds long-chain fatty acids via zDHHC acyltransferases and is reversed by the thioesterases APT1 and APT2, which control receptor trafficking, vesicle fusion, and compartmentalized signaling. Lysine acylations—including malonylation, succinylation, and crotonylation—use acyl-CoA donors, are written by acyltransferases, and are erased mainly by sirtuins (Figure 8). This couples these marks to acyl-CoA and NAD^+^. Across metabolic tissues, acylation regulates chromatin and mitochondrial pathways and modulates transporter localization, enzyme activity, insulin secretion and response, and lipid homeostasis [174,175].

Palmitoylation is the reversible attachment of palmitate to cysteine residues and regulates receptor maturation, signaling, and membrane trafficking in metabolic tissues. Early biochemical work showed that insulin and IGF-1 receptors carry covalently bound palmitate on the receptor beta subunit. This finding suggested roles for S-acylation in receptor maturation and signaling [176]. Palmitoylation supports insulin secretion and receptor trafficking in pancreatic beta cells. SNAP-25 requires cysteine palmitoylation for plasma membrane targeting and efficient exocytosis [177]. GLP-1 receptor agonists induce C-terminal palmitoylation, which promotes nanodomain clustering and endocytosis, and modulators of this process alter insulin secretion [178]. APT1 (acyl-protein thioesterase 1) is a cytosolic thioesterase that depalmitoylates S-acylated proteins and terminates palmitoylation cycles. In pancreatic beta cells, APT1-dependent depalmitoylation restrains palmitoylation-driven exocytosis, and APT1 deficiency in human islets and mouse models causes insulin hypersecretion followed by progressive beta cell failure [179]. In adipocytes, palmitoylation is a key regulator of signaling, differentiation, and fatty acid uptake. AKT palmitoylation at Cys344 supports phosphorylation and is required for adipocyte differentiation [180]. Dynamic palmitoylation controls CD36 trafficking and function in adipocytes and couples membrane residency and endocytosis to fatty acid uptake. DHHC4 and DHHC5 maintain this cycle from distinct compartments, and disruption of palmitoylation reduces adipocyte fatty acid uptake and limits lipid accumulation [181,182]. In addition, palmitoylation also regulates glucose transporter trafficking and insulin signaling complexes in adipocytes. DHHC7 palmitoylates GLUT4 at Cys223 and is required for insulin-stimulated GLUT4 membrane translocation and glucose uptake [183]. Insulin drives a caveolin-2 palmitoylation cycle via APT1 and ZDHHC21 that organizes IR–Cav-2–IRS1–AKT signaling and promotes glucose uptake and lipogenesis [184].

Crotonylation is a lysine acylation that uses crotonyl-CoA as a donor and is enriched on histones where it regulates transcriptional activity. The mark is reversible and is recognized by reader modules, linking metabolic state to chromatin function. SIRT1, SIRT2, and SIRT3 function as histone decrotonylases, and SIRT3 has been shown in cells to regulate histone crotonylation and gene expression [185,186]. In beige adipocytes, HDAC1 removes H3K18 crotonylation at Pgc1a and Ucp1 enhancers and promoters, lowering thermogenesis, whereas HDAC1 inhibition enriches crotonylation and enhances energy expenditure in vivo [187].

SIRT5 is an NAD^+^-dependent lysine desuccinylase and demalonylase that removes acidic acylations from mitochondrial proteins and establishes sirtuins as regulators of non-acetyl acylations [188]. Proteome-wide analyses in mouse liver show widespread lysine malonylation that increases with Sirt5 deficiency. Glycolysis is a principal target, and Sirt5 loss reduces glycolytic flux [189]. Additionally, diabetic mouse liver shows broad lysine malonylation across enzymes involved in glycolysis, gluconeogenesis, and lipid metabolism [190]. In parallel, SIRT5 acts as the predominant mitochondrial desuccinylase. Proteome-wide analyses show that lysine succinylation is widespread and regulated by SIRT5. SIRT5 desuccinylates subunits of the pyruvate dehydrogenase and succinate dehydrogenase complexes and modulates mitochondrial respiration [191]. In liver, SIRT5 also targets HMGCS2, and lysine-to-glutamate mutation at hypersuccinylated sites reduces its activity, which supports a role for SIRT5 in hepatic ketogenesis [192]. In brown adipose tissue, SIRT5 restrains mitochondrial succinylation and malonylation, with UCP1 as a key substrate. BAT-specific Sirt5 loss increases acylation, destabilizes UCP1, impairs respiration and mitophagy, and reduces thermogenic capacity [193].

### 4.7. SUMOylation

Small ubiquitin-like modifier (SUMO) conjugation is a reversible post-translational modification in which SUMO proteins are covalently attached to specific lysine residues of target substrates. This modification alters protein stability, controls intracellular trafficking, and regulates transcriptional activity. The conjugation reaction proceeds through a hierarchical cascade of E1-activating, E2-conjugating, and E3-ligating enzymes, whereas SUMO-specific proteases (SENPs) remove SUMO to preserve dynamic balance. Through this regulated cycle, SUMOylation modulates essential cellular pathways such as transcriptional regulation, DNA damage repair, cell-cycle progression, and stress responses (Figure 7F). In metabolic tissues, SUMOylation functions as a nutrient- and stress-sensitive hub that coordinates hormonal signals with transcriptional programs and metabolic homeostasis [194,195,196].

In the liver, SUMOylation integrates nutrient signals with lipid metabolism and fasting responses. Dysregulation of this process accelerates lipogenesis and MASLD progression. PIASy-mediated SUMOylation of SREBP-1c promotes its degradation and suppresses lipogenesis, whereas PIASy loss enhances hepatic steatosis [197]. Nutritional stress–induced deSUMOylation of FoxA1 reduces Sirt6 expression and fatty-acid oxidation, accelerating steatosis [198]. SUMOylation of Prox1 functions as a nutrient-sensitive switch that declines during fasting but is blunted in obesity, and hepatocyte-specific knock-in of a SUMO-deficient Prox1 lowers systemic cholesterol [199]. In contrast, defective SUMOylation of LRH-1 under lipogenic conditions activates lipogenic programs and exacerbates MASLD [200].

Adipose SUMOylation regulates pathways essential for maintaining systemic insulin sensitivity under nutritional stress. SENP2 promotes adipogenesis and lipid storage by counteracting repressive histone marks, preserving lipid-storage capacity and preventing ectopic lipid accumulation. Loss of SENP2 reduces adipogenic potential and predisposes to insulin resistance under high-fat diet conditions [201]. UBC9-dependent SUMOylation of ER proteins, including ERp44, aggravates ER stress and metabolic dysfunction, whereas disruption of this modification alleviates stress and protects against diet-induced insulin resistance [202]. In thermogenic adipose depots, SENP2 promotes brown adipocyte differentiation by suppressing Necdin and limits browning of white adipose tissue by deconjugating SUMO from C/EBPβ [203,204].

In pancreatic beta cells, SUMOylation functions as a central regulatory mechanism linking transcriptional control, stress adaptation, and survival. It stabilizes key regulators of the insulin gene and maintains the balance between conjugation and deconjugation. PDX1, a master regulator of beta cell identity and insulin transcription, is SUMOylated by SUMO-1, which promotes nuclear retention and enhances insulin transcriptional activity [205]. MafA, a beta-cell–specific transcription factor required for glucose-responsive insulin expression, is SUMO-1/2–modified at Lys32. This modification increases under low glucose or oxidative stress and reduces insulin promoter activation [206]. Ubc9, the sole E2-conjugating enzyme in the SUMO pathway, transfers SUMO to substrates and is essential for beta cell homeostasis. Its deletion triggers ROS-driven beta cell death and diabetes, whereas overexpression enhances NRF2-dependent antioxidant defense but suppresses insulin secretion [207]. Enhanced SUMOylation protects beta cells from IL-1β-induced apoptosis, reduces iNOS expression, caspase-3 cleavage, and NF-κB nuclear entry. Conversely, SENP1 overexpression impairs insulin secretion and promotes beta cell apoptosis [208].

Beyond transcriptional regulation and survival, SUMOylation modulates key steps in GSIS. SUMO-1 modification of the voltage-gated K^+^ channel Kv2.1 reduces conductance and membrane excitability, an effect enhanced by Ubc9 and reversed by SENP1 [209]. SUMOylation suppresses post-docking insulin exocytosis in rodent and human beta cells, and deSUMOylation reverses this effect to promote secretion [210,211]. The isocitrate–SENP1 pathway amplifies glucose-stimulated insulin exocytosis [212]. SUMOylation also regulates beta cell metabolic sensing, as glucokinase modification increases its activity and stability [213], and SENP2-mediated deSUMOylation of DRP1 preserves mitochondrial function to sustain GSIS [214]. Importantly, the incretin effect in pancreatic beta cells is regulated by SUMOylation. GLP-1R–driven cAMP generation and insulin secretion are attenuated by SUMO-1, whereas SENP1 is required for incretin-enhanced exocytosis and for maintaining oral glucose tolerance under metabolic stress [215,216].

SUMOylation maintains metabolic homeostasis by regulating hepatic lipid metabolism, insulin secretion in beta cells, and lipid storage and thermogenesis in adipose tissue. Dysregulation of this pathway contributes to insulin resistance and MASLD, establishing SUMOylation as a promising therapeutic target in metabolic disease.

### 4.8. S-Nitrosylation

S-nitrosylation is a reversible, redox-dependent post-translational modification in which a nitric oxide (NO) group covalently attaches to specific cysteine residues to form S-nitrosothiols. Cellular S-nitrosylation is regulated by nitric oxide synthases, protein-to-protein transnitrosylation, and denitrosylating systems such as GSNOR and thioredoxin (Figure 7G). It modulates protein conformation, activity, localization, and interactions in a site-specific manner and links oxidative signaling to core metabolic pathways. In metabolic tissues, S-nitrosylation intersects with insulin signaling and nutrient homeostasis [217,218].

S-nitrosylation disrupts key processes of hepatic insulin regulation and metabolic homeostasis. S-nitrosylation of the insulin-degrading enzyme (IDE) inhibits its catalytic activity and impairs cellular insulin degradation [219]. SCAN (biliverdin reductase B) functions as a protein S-nitrosylase that uses S-nitroso-CoA to selectively nitrosylate the insulin receptor and IRS1, and this modulates insulin signaling [220]. Obesity-induced iNOS promotes S-nitrosylation of IRE1α, which suppresses XBP1 splicing and impairs ER function [221]. Hepatic dysfunction of the denitrosylase GSNOR elevates S-nitrosylation of lysosomal enzymes, disrupts autophagic flux, and promotes insulin resistance in obesity, whereas restoring GSNOR activity or expressing nitrosylation-resistant lysosomal proteins rescues autophagy and improves insulin action [222].

S-nitrosylation is a key mechanism linking nitric oxide signaling to insulin resistance in skeletal muscle. Acute exercise lowers iNOS and reverses S-nitrosylation of IRβ, IRS1, and AKT, and it restores early insulin signaling and insulin sensitivity in diet-induced obese rats [223]. S-nitrosylation of AKT at Cys224 suppresses kinase activity and is elevated in db/db mice [224]. LPS-induced iNOS drives nitrosylation of IRβ, IRS1, and AKT, which blunts insulin-stimulated phosphorylation and causes insulin resistance [225]. Proteomic analysis reveals nearly 500 nitrosylated cysteine sites across mitochondrial, contractile, and metabolic proteins. These widespread modifications cluster in pathways such as the TCA cycle, glycolysis, glutathione metabolism, and fatty acid oxidation, and they demonstrate that S-nitrosylation extends beyond insulin signaling to encompass core energy and redox networks in skeletal muscle [226].

In adipocytes, obesity increases protein S-nitrosylation of IRβ and AKT and is accompanied by increased iNOS and decreased thioredoxin reductase. S-nitrosylation of PDE3B at Cys768 and Cys1040 reduces insulin-stimulated PDE3B activation and weakens the anti-lipolytic action of insulin [227]. In endothelial cells, nitric oxide promotes transendothelial insulin transport and enhances tissue delivery. S-nitrosylation of protein tyrosine phosphatases such as PTP1B and SHP2 sustains endothelial insulin-receptor signaling and facilitates insulin uptake and transendothelial transport [228,229]. S-nitrosylation also operates in the nervous system to regulate central insulin action and energy balance. Obesity induces hypothalamic iNOS and S-nitrosylation of IRβ and AKT, which impairs central insulin signaling and disrupts energy balance [230].

S-nitrosylation directly modulates beta cell glucose sensing and insulin secretion. Insulin elevates nitric oxide (NO) and nitrosylates glucokinase at Cys371, changing its conformation and subcellular localization [231]. GLP-1 enhances GSIS by inducing glucokinase S-nitrosylation at Cys371, which increases enzymatic activity and promotes release from secretory granules [232]. Glucose triggers rapid S-nitrosylation of syntaxin-4 at Cys141 in human islets and MIN6 beta cells within minutes, which promotes VAMP2 binding and facilitates insulin granule exocytosis [233].

### 4.9. Neddylation

Neddylation is a reversible ubiquitin-like modification in which NEDD8 is conjugated to lysine residues, predominantly on cullins, by the NEDD8-activating enzyme NAE1–UBA3, the E2 conjugases UBE2M and UBE2F, and E3 ligases such as RBX1 or RBX2. Deneddylation is mediated by the COP9 signalosome through its metalloprotease subunit CSN5 (Figure 7H) [234]. Recent findings demonstrate that neddylation plays important roles in metabolic regulation. In the liver, neddylation regulates glucose homeostasis at multiple nodes. Inhibition of cullin neddylation stabilizes insulin receptor substrates, enhances hepatic insulin signaling, and lowers glucose production [235]. Fasting induces neddylation of phosphoenolpyruvate carboxykinase 1, which increases enzymatic activity and sustains gluconeogenesis, whereas disruption of this modification reduces hepatic glucose output and improves glycemic control [236]. In obesity, macrophage UBE2M-mediated neddylation of TRIM21 promotes VHL degradation, stabilizes HIF-1α, and elevates IL-1β, driving inflammation and metabolic dysfunction. Deletion of macrophage Ube2m or TRIM21 antisense therapy alleviates insulin resistance and hepatic steatosis [237].

## 5. Conclusions

Post-translational modifications function as integrated regulatory layers that connect metabolic inputs to transcriptional and signaling outputs across metabolic organs. These mechanisms define tractable targets for pharmacology and genetic intervention and support biomarker development that reflects pathway engagement. In type 2 diabetes, dysregulated PTM networks contribute to insulin resistance in peripheral tissues and to beta cell dysfunction, which links PTM biology directly to disease initiation and progression.

Key challenges remain. The complexity of PTM crosstalk complicates target selection and demands multiplexed readouts with temporal and spatial resolution. Precise characterization of PTM changes in defined cell types and disease stages will be crucial for the development of therapies that restore metabolic homeostasis without disrupting essential adaptive responses. Single-cell and spatial proteomics, quantitative PTM stoichiometry, and integrative multi-omics are needed to capture heterogeneity across tissues and disease stages. Chemical probes and genetic tools that selectively perturb writers, erasers, and readers will clarify pathway logic and limit off-target risk. Translational progress will benefit from human tissue-anchored studies, longitudinal cohorts with PTM biomarkers, and patient stratification that aligns pathway activity with therapeutic choice. Research in this area has the potential to identify interventions that can protect insulin secretion and insulin response organs from metabolic and inflammatory stress and to improve glycemic control in individuals with metabolic syndrome and diabetes.

## Data Availability

No new data were created or analyzed in this study.

## Abbreviations

The following abbreviations are used in this manuscript:

AGEs	Advanced Glycation End products
AKT	Protein Kinase B
AMPK	AMP-activated Protein Kinase
APT1	Acyl-protein thioesterase 1
APT2	Acyl-protein thioesterase 2
CARM1	Coactivator-associated arginine methyltransferase 1
CD36	Cluster of Differentiation 36
ChREBP	Carbohydrate-Responsive Element-Binding Protein
CTCF	CCCTC-binding factor
DHHC7	Zinc Finger DHHC-type Palmitoyltransferase 7
eIF2a	Eukaryotic Translation Initiation Factor 2 alpha
EZH2	Enhancer of zeste homolog 2
FAS	Fatty Acid Synthase
FOXO	Forkhead box O
GK rats	Goto-Kakizaki rats
GLUT4	Glucose Transporter Type 4
GSIS	Glucose-Stimulated Insulin Secretion
GSK3	Glycogen Synthase Kinase 3
HATs	Histone Acetyl-Transferases
HCC	Hepatocellular Carcinoma
HDACs	Histone Deacetylases
HMGA1	High Mobility Group A1
IGF-1	Insulin-like growth factor 1
IRS-1	Insulin Receptor Substrate 1
KDM1A/B	Lysine Specific Histone Demethylase 1A/B
LncRNA-NEAT1	Nuclear Paraspeckle Assembly Transcript 1
MafA	v-maf musculoaponeurotic fibrosarcoma oncogene homolog A
MARK2	MAP/Microtuble Affinity-Regulating Kinase 2
MARK3	MAP/Microtuble Affinity-Regulating Kinase 3
MASLD	Metabolic Dysfunction-Associated Steatotic Liver Disease
MINDY1	MINDY Lysine 48 deubiquitinase 1
MIP-CreERT	Mouse Insulin 1 Promoter-Cre-ERT
MLL4	Mixed-lineage leukemia 4
mTOR	Mechanistic Target of Rapamycin
NR4A1	Nuclear Receptor Subfamily 4, group A, Member 1
OGT	O-GlcNAc Transferase
PDE3B	Phosphodiesterase 3B
PDX1	Pancreatic and Duodenal Homeobox 1
PERK	Protein Kinase R-like ER Kinase
PGC1-a	Peroxisome Proliferator-activated receptor gamma coactivator 1- alpha
PKA	Protein Kinase A
PKC	Protein Kinase C
PNPLA3	Patatin-like phospholipase domain-containing 3
PPARγ	Peroxisome Proliferator-Activated Receptor gamma
PRMTs	Protein Arginine Methyltransferases
PTM	Post Translation Modification
RIP-Cre	Rat Insulin Promoter-Cre
RNF20	Ring Finger Protein 20
Rpd3	Reduced Potassium Dependency 3
SCAN	Biliverdin Reductase B
SCD	Stearoyl-CoA Desaturase
SENP1	SUMO-specific peptidase 1
SETD7	SET domain-containing lysine methyltransferase 7
SIRTs	Sirtuins
SNAP-25	Synaptosome-associated Protein 25
SREBP1c	Sterol Regulatory Element-Binding Protein 1c
TGF-β	Transforming Growth Factor beta
TNFα	Tumor Necrosis Factor alpha
TSC2	Tuberous sclerosis complex 2
UCP-1	Uncoupling Protein 1
ULK1	Unc-51 Like Autophagy Activating Kinase 1
UPR	Unfolded Protein Response
USP2A	Ubiquitin-specific Protease 2a
VAMP2	Vesicle-associated membrane protein 2
VEGFA	Vascular Endothelial Growth Factor A
zDHHC	Zinc fingers DHHC-type
ZDHHC21	Zinc Finger DHHC-type Palmitoyltransferase 21
